# Obesity in International Migrant Populations

**DOI:** 10.1007/s13679-017-0274-7

**Published:** 2017-07-28

**Authors:** Marie Murphy, Wendy Robertson, Oyinlola Oyebode

**Affiliations:** Warwick Medical School, University of Warwick, Gibbet Hill Campus, Coventry, CV4 7AL UK

**Keywords:** Obesity, Migration, Ethnicity, Epidemiology

## Abstract

**Purpose of Review:**

This review examines the risk of obesity in migrant groups—specifically migrants from countries with lower prevalence of obesity to countries with higher prevalence of obesity. We examine obesity prevalence within migrant groups compared with native populations and the evidence on factors that might shape obesity risk in these migrant groups.

**Recent Findings:**

Migrants may arrive in new countries with a health advantage including generally a healthier body weight. Genetic and epi-genetic factors, as well as body size preference, socio-economic factors, and stress exposure, may play a role in increasing unhealthy weight gain in migrant populations. This unhealthy weight gain leads to similar or greater obesity risk in migrant populations compared with native populations 10–15 years after migration.

**Summary:**

Meeting the challenge of prevention and treatment of obesity in diverse populations will require greater attention to minority groups in research in the future.

## Introduction

In 2010, overweight and obesity were estimated to have caused 3.4 million deaths and 94 million disability-adjusted life-years worldwide [1]. Worldwide, prevalence of obesity is rising: a systematic analysis for the Global Burden of Disease study examining data on prevalence of overweight and obesity in children and adults from 1980 to 2013 found that overweight and obesity (based on a body mass index (BMI) over 25 kg/m^2^) had risen by 27.5% for adults and 47.1% for children—that is, an increase from 921 million individuals in 1980 to 2.1 billion in 2013 [2•]. While this trend is monotonic in populations across the world, the prevalence and risk of obesity vary between and within populations [2•]. It is likely that the variation in prevalence and risk of obesity is due to genetic (including ethnic) differences between populations as well as due to the variation in the degree to which local environments are ‘obesogenic’.

This review examines the risk of obesity in migrant groups, focusing on international migration which is often from regions with low prevalence of obesity to regions with higher prevalence of obesity. In this review, we include ‘immigrant’ populations within our examination of migrants i.e. those who are settled in their new country, and we will also consider subsequent generations whose parents or grandparents were migrants, wherever possible considering these subsequent generations as distinct groups. Examination of this topic will often involve referring to ethnic groups—groups of people with shared ancestry. We note that any research or discussion of ‘the migrant population’ will involve some generalisations, and that the migrant population is not homogenous and neither are the many ethnic groups that are often classified based on convenience (e.g. for the sake of sample size, amalgamating people from diverse backgrounds, traditions and cultures as ‘black’, ‘white’ and ‘Asian’).

Many differences between the health of migrant and non-migrant populations have been identified (better and worse), some of which arise from inequalities [3•]. In Europe, in general, migrants are reported to be more vulnerable to communicable diseases, occupational hazards and poor mental health, as well as diabetes and perinatal morbidity [4]. In the USA, migrants live 3.4 years longer than the US-born population, and an advantage in life expectancy remains when stratified by ethnic group [5]. Migrants to the USA are less likely to have a disability, but are more likely to be diagnosed with HIV, have low levels of health insurance coverage and have reduced access to preventative health services [5].

Migration itself is an important determinant of health—due to the economic, physical and mental burden of uprooting and travelling to start life somewhere new [3•]. There are certainly many other factors that might shape obesity risk associated with migration status including intrinsic factors related to the genetic background of individuals, lifestyle factors related to culture and the wider determinants of health including education, occupation and more. Indeed, it is also likely that obesity risk changes with time of residence in the host country. We examine obesity prevalence within migrant groups compared with native populations, and the evidence on factors that might shape obesity risk in these migrant groups.

### Obesity Prevalence

There are two key reasons why we might expect obesity prevalence to be lower in migrants than in native populations [6]. Firstly, migration is stressful and expensive, it can include passing rigorous health checks and, as recently as 50 years ago, long journeys could be physically demanding. For these reasons, migrants (individuals with the capacity to overcome these challenges) are usually healthier than both the population they leave behind and the one they join (the ‘healthy migrant effect’), with the exception of asylum seekers [3•]. Secondly, destination countries are often those that have reached the stage in the nutrition transition in which there has been increasing fat, sugar and processed food consumption, while source countries are often earlier in this transition. This means that even if migrants were randomly selected from source populations, rather than a healthier self-selecting group, they would be likely to have a lower BMI on arrival than the native population (although note that obesity is increasing worldwide and in some middle-income countries it is particularly high e.g. Mexico).

Consistent with the hypothesis above, there is evidence that newly arrived migrants have a greater prevalence of healthy weight, than native populations. A cross-sectional study of over 250,000 people from the six largest Asian ethnic groups resident in the USA found that after adjustment for age and ethnicity, US-born men had 4× the odds of being obese than migrant men, and for women the odds were 3.5× [7]. Similar studies of other migrant populations have reported similar findings [8–11].

Despite this initial advantage, unhealthy weight gain by migrants generally increases substantially so that 10–15 years post migration, overweight and obesity rates approach or overtake those of the native population [6, 12]. There has been debate about cohort effects confounding this relationship. This was addressed in a study of six year-of-entry cohorts of immigrants to the USA, which demonstrated that while self-rated health actually improved with a longer duration of residence after accounting for cohort effects, obesity prevalence showed the opposite pattern [13].

The pattern for children is likely to be similar, with slimmer newly arrived migrants, and later unhealthy weight gain. An analysis of the US National Health and Nutrition Examination Survey (NHANES) 1999–2012 found that obesity prevalence was significantly higher in US-born children/adolescents than in those who had been in the USA for under a year; however, this gap decreased from 1999 to 2011 due to a rapid increase in obesity prevalence among the non-US-born population [14]. A systematic review of prevalence of overweight and obesity among European child populations reported that migrant children are at higher risk for overweight and obesity than children from native populations [15]. However, most included studies reported differences by ethnic group, or classified children according to their mother tongue, or parent’s country of birth so this is likely to have captured more individuals from subsequent generations than it did true migrant children themselves. Another study suggests that some children of migrants gain weight faster than those with US-born parents [16].

We have discussed why migrant populations are likely to arrive with lower average prevalence of overweight and obesity than resident populations in high-income countries (HICs); however, a number of factors might combine to make weight gain likely in migrant populations after arrival in a new country and in subsequent generations. These will be discussed in turn below, and see Table 1.

**Table 1:** Factors associated with risk of unhealthy weight gain in migrant populations

Factor	Conclusion
Genetic and physiological factors	Likely that some genetic differences underlie vulnerability of different ethnic groups to obesity. No candidate genes yet identified.
Epi-genetic factors	Likely that migrant populations are more often predisposed to increasing obesity risk when exposed to an obesogenic environment.
Dietary behaviour	Although migrants may have an increasingly unhealthy diet after settling in a HIC, their diet is generally healthier than native populations so this is unlikely to be a major contributor to increased obesity risk.
Physical activity	Migrants generally do more occupational physical activity and physical activity for travel, but face barriers to engaging in leisure-time physical activity. Reduced leisure-time physical activity may become an increasingly important determinant of increased obesity risk in the future.
Body size preference	Preference or tolerance of larger female body sizes is changing worldwide. This may currently increase obesity risk through reduced motivation to maintain or lose weight in some migrant groups.
Acculturation	In general migrants who maintain their original culture have reduced obesity risk.
Socio-economic status	It is likely that social and economic disadvantage increase obesity risk in migrants, as with other population groups in HICs.
Stress	It is plausible that stress experienced by migrants increases obesity risk.

### Genetic and Physiological Factors

Animal and family studies have demonstrated that some of the variation in body composition is likely to have a genetic component [17]. Several hormones are involved in signalling satiety and promoting appetite including leptin, adiponectin and insulin, and production of these molecules is under genetic control. About 127 sites in the human genome have been reported to link with development of obesity [18].

Given that ethnic groups share common ancestry and therefore have genetic similarities, it is possible that there are some ethnic groups that are more prone to development of obesity than others. There are some general differences in physiology between groups defined by ethnicity, which could be under genetic control. For example, a study examining healthy lean South Asian and white adults in the Netherlands found that South Asian adults have lower resting energy expenditure, non-shivering thermogenesis and brown adipose tissue volumes which might underlie high susceptibility to obesity and metabolic disorders [19]. In a study of Asian Indian, Creole and white Swedish men and women, Asian Indians of both sexes and Creole men had higher leptin levels than white Swedish men and women [20].

Some historical migrant populations may have been shaped by exposure to particularly challenging journeys. This may have applied a selective pressure to the populations in which only those individuals capable of surviving tough conditions completed the journey to pass on genetic material to subsequent generations. The example of Africans who travelled to the USA during the slave trade is an extreme example of this.

Researchers have examined whether genetic differences might underlie differing vulnerability to obesity and metabolic disorders between ethnic groups e.g. [21]. However, despite this being theoretically possible, to our knowledge, genes that might be responsible have not been identified.

### Epi-genetic Factors

Sometimes known as the ‘Barker Hypothesis’ (after the proponent of the theory) or the ‘Thrifty phenotype’, the idea that susceptibility to adult disease can be programmed in the womb has been supported by human and animal studies [22, 23]. According to this theory, an adverse environment in the prenatal and perinatal period can programme the foetus to have a particular metabolic profile in later life. Specifically poor maternal nutrition can programme the foetus to be more likely to store calories as fat, and have elevated fasting blood sugar. While this may have been evolutionarily advantageous, because the individual is metabolically prepared for an environment in which food is scarce, in the case that there is under-nutrition in the womb and then later exposure to a calorie-rich, fat-rich, sugar-rich diet, this backfires and the individual becomes vulnerable to obesity and other adult diseases. The percentage of babies born with a low birth weight (one marker of insufficient nutrition in the womb) is particularly high in low- and middle-income countries (LMICs) at 27% of live births [24]. This has been postulated as the cause of increased overweight and obesity in international adoptees living with Swedish families in Sweden [25] and for the differences between British-born and migrant UK Pakistani women in a very small study that found British-born women were taller and had healthier metabolic markers than the Pakistani-born women [26]. In a sample of 337 sub-Saharan African children who had migrated to Australia, higher prevalence of overweight and obesity was associated with lower birth weight [27]. It is clear that migrants from LMICs to HICs may be more likely to have had poor nutrition prenatally or in early life, which may then predispose these individuals to obesity when in the HIC’s obesogenic context.

### Cultural Factors

#### Dietary Behaviour

Dietary behaviour varies widely among and within different cultures. When people migrate, dietary practices are often disrupted, so that different types of food are consumed or preparation of food is modified [28]. Of course, migrants also frequently influence the tastes of native populations, increasing the range of foods on offer in host countries.

A systematic mapping of 37 primary studies examining dietary behaviour in minority ethnic groups across Europe found that factors influencing dietary behaviour included cultural identity, religious beliefs and prescribing, and taste preferences [29•]. Specific factors influencing dietary behaviour identified for migrant populations were availability of traditional foods (noting that when these are imported they can be more expensive), competency in new language and perception of host culture (including eating behaviours and food habits) [29•].

A review of dietary habits of minority ethnic groups in Europe reports that the majority of migrants combine part of their traditional diet with some of the less healthy elements of the European diet [28]. Usually, the staple foods remain (such as rice, breads, noodles) while more processed foods are consumed in addition, particularly by younger generations (such as breakfast cereal, soft drinks and high-fat high-sugar snacks) [28]. A survey of 599 Mexican-origin women found that US-born, Mexican-origin women consumed more sugar-sweetened beverages and more fast-food meals than Mexican-born, Mexican-origin women [30]. As well as increasing consumption of unhealthy foods associated with the diet of the new country, there is a theory that foods traditionally eaten at festivals (which have a higher calorie, sugar and fat content than other traditional foods) which would have been eaten a few times a year on special occasions become part of the new daily diet of settled migrants, with supporting data from the USA and UK [31]. In addition, a qualitative research study with 25 Pakistan-born women living in Oslo, Norway, revealed an increasing social pressure to be a good host with an expectation of serving an abundance of different dishes to honour guests [32].

Despite the increased consumption of unhealthy items in migrants who settle in a new country, evidence from both the USA and UK suggests that dietary practices are still generally healthier in migrant than native populations. For example, analysis of NHANES data 2007–2008 demonstrated that US households with a foreign-born reference person cooked more dinners at home (mean of 5.8 per week) than households with a US-born reference person (mean of 4.9 per week) which is usually a marker of better diet quality [33]. Mexican Americans born in Mexico have higher consumption of fruit and vegetables, and a higher overall percentage of energy coming from healthy food sources than non-Hispanic White Americans or Mexican Americans born in the USA [34].

In the UK, large health survey data suggests that minority ethnic groups have some elements of a healthier diet than the white British population including a larger proportion who meet the recommended five portions of fruit and vegetables a day, particularly within Chinese and African-Caribbean groups, and generally lower fat intakes [35]. Breast-feeding rates are also higher for minority ethnic groups [35], and there is lower alcohol consumption in Asian, black Caribbean and black African ethnic groups living in the UK [36].

There are exceptions to the generally healthier diet. One example from the USA is that Mexican-born women (non-pregnant, aged 15–44 years) have lower serum folate compared with US-born Mexican women regardless of supplement use [37]. In the UK, those from minority ethnic groups report using more salt [35]. Migrant groups in the Netherlands are less likely to have breakfast than Dutch-born groups [38]. However, given that overall, there is some evidence that diet in migrant groups is healthier than that of native populations, it is unlikely that diet alone is responsible for the increasing risk of obesity in settled migrant populations.

#### Physical Activity

Age-standardised prevalence of insufficient physical activity in adults aged 15+ years presented by the WHO, based on data from 2008, showed the highest rates of physical inactivity in high-income countries, with upper middle-income countries very close behind and lower rates of inactivity in lower middle- and low-income countries [39]. In terms of the WHO region, the Americas and Eastern Mediterranean regions had the highest rates of inactivity (approaching 50% of women and 40% of men). The lowest rates on inactivity were seen in the African (approximately 30% of women and 25% of men) and South-East Asian regions (approximately 20% for women, 15% for men) [39]. This suggests that migrants from LMIC to HIC will be moving from more physically active populations to less physically active populations, in general.

Analysis of objectively measured physical activity data from NHANES showed that there were significant differences in the amount of moderate to vigorous physical activity achieved by different population groups delineated by ethnicity and migrant status in the USA. Migrant white and Hispanic groups and the female migrant-black group achieved more physical activity than their US-born counterparts, but for male black groups, this was reversed [10]. Another NHANES study showed that US-born Mexican Americans engaged in more minutes per week of leisure time physical activity relative to Mexican-born counterparts. However, US-born individuals were also more likely to report having done no work or travel-related physical activity and therefore reported fewer minutes of total physical activity [40]. For children, analysis of the US National Survey of Children’s Health showed that migrant children were substantially less likely than native-born children to engage in sports and physical activity [5] which was reflected in analysis of the Canadian Health Behaviour in School-Aged Children Study [41].

There are a number of barriers to participation in leisure time physical activity experienced by migrant groups. One of the main barriers reported in the literature is a restriction on opportunity for women to take part in physical activity primarily due to religious dress codes. In societies set up to cater for this, there may be women-only settings in which women are able to exercise—but on migration to a new society, these opportunities are reduced. In the UK, the gap between male and female participation in sport is greater among minority ethnic groups than in the population as a whole [42].

Other barriers to participation in sport in UK minority ethnic groups, including migrant groups, are overt racism (e.g. name-calling), cultural factors (e.g. less acceptable for children to go to a park without their parents) and factors which may be related to socio-economic status, which will be discussed in more detail below (e.g. fear of attacks or mugging and poor access to or condition of leisure facilities) [42]. In a study of Somali women living in Sweden, climate was also considered a barrier to an active lifestyle [43].

It seems likely that migrants generally do more occupational physical activity and physical activity for travel and less leisure time physical activity than native populations. This may change as fewer jobs require physical activity [44], and it seems likely that motorisation of transport will continue. In this scenario, leisure time physical activity may become a more important determinant of rates of obesity in migrant groups.

#### Body Size Preference

There has been much research examining whether different cultures have a different preference in terms of body size, particularly of women. Where food is scarce, higher body fat may be seen as a sign of high status, and considered preferable for survival and reproduction [23]. It is difficult to determine which population groups value larger body sizes—for example black women in the USA tend to be heavier than their white counterparts and express less dissatisfaction with their weight; however, this may be due to higher overall self-esteem among black women [45]. A study of more than 1200 men and women living in the USA found that ethnicity had no influence on preference for female or male shapes or tolerance for obesity [45]. In a questionnaire study, the majority of Black Somali mothers living in the UK felt that being overweight in childhood can be healthy, which was considerably higher than parents from other ethnic backgrounds; however, a similar pattern was not observed in the broader ‘Black African’ group in this sample [46]. A systematic review of beauty ideals and body dissatisfaction in Africans living in Africa and Europe found that ethnic groups living in greater isolation or with low incomes prefer a larger body shape, but that those in urban areas and in Europe have increasing BMI and body dissatisfaction [47]. A qualitative study of Moroccan-born women in the Netherlands and in Morocco found that all participants reported their preferences shifting to slimmer body sizes but that weight gain was still seen as a sign of success [48]. It is likely that migrant groups from LMICs in general have a preference or a tolerance of larger female body sizes than HIC populations, which might influence desire to maintain or lose weight. However, this may well be changing.

#### Acculturation

Migrants are immersed in their host environment and undergo acculturation to some degree, i.e. changing attitudes and behaviour under the influence of the host culture. A systematic review of evidence on the relationship between acculturation and overweight/obesity among adult migrants from LMICs to HICs identified nine studies and reported that for men, acculturation promotes unhealthy weight gain, but for women there were inconsistent results [12]. This suggests that in general, migrants who maintain attitudes and behaviour associated with the country of origin are protected from the increasing obesity risk. In this review, the authors hypothesise that for some women, the adverse dietary and physical activity changes associated with the new environment are off-set by the social norms in many HICs which value slim female bodies. This hypothesis is supported by one of the included studies in which obese Mexican Americans with low levels of acculturation were more satisfied with their weight and body shape than those who were highly acculturated [12].

### Wider Determinants of Health

#### Socio-economic Status

It is well documented that there is a link between better socio-economic status and better health [49]. Systematic reviews report that for adults and children in HICs, the least disadvantaged groups are less likely to be obese while in low-income countries there is a positive relationship between socio-economic status and obesity, which reverses with increasing economic development [50–53]. There are likely to be many mediators of the relationship between socio-economic status and obesity including knowledge of the aetiology of disease and causes of obesity, as well as barriers associated with money, time and opportunities (e.g. within a local neighbourhood) to eat a healthy diet or take part in physical activity.

Migrants tend to settle in areas with cheap housing and are more likely to hold jobs which are less prestigious, to have poorer job security and to endure more stressful working conditions including working unsociable hours [54]. Subsequent generations generally demonstrate significant upward social mobility, although some groups experience marginalisation and reduced opportunities [55]. Even when migrant groups have similar current incomes, there can be large differences in education levels, material resources (e.g. property ownership) and accumulated wealth [56].

Although many studies examine the contribution of socio-economic status to differences in obesity by ethnic group, very few consider migration status. In terms of ethnicity, studies have reported that deprivation does affect the engagement of minority ethnic groups in physical activity [42], but differences in children’s obesogenic lifestyles between ethnic groups in the UK were not explained by deprivation [57]. Where migration status is examined, results are mixed. For example, in a study examining changes in waist circumference over time in Chinese and Hispanic immigrants to the USA, there was some evidence that living in neighbourhoods more supportive of healthy behaviour (specifically walkability and recreational resources both of which are associated with more expensive neighbourhoods) tempered the increases in waist circumference in the Chinese group, but not the Hispanic group [58]. The lack of findings on socio-economic status and unhealthy weight gain in migrant populations does not mean this is not an important mediator; it is more likely challenges in measuring socio-economic status (particularly in migrants) which makes this difficult to study.

#### Stress

Migration may be associated with a lack of social support in the new country, social isolation (due to language, cultural or administrative barriers) and sometimes experiences of marginalisation or racism. These factors mean that some migrants are likely to experience some degree of stress. Stress has been linked to obesity [59, 60], for example through an association between stress and increased food intake, as well as potentially physiological effects of stress on metabolism [61, 62].

A meta-analysis of 293 studies found that experience of racism is significantly related to poorer health [63]. When obesity-related outcomes including BMI, waist circumference and waist-to-hip ratio were examined specifically, these were also significantly associated with racism. A third of included studies examined migrant populations, although the majority were conducted with native-born populations in the USA [63]. A systematic review identified 32 studies that found racism had a negative impact on the involvement and experiences of minority ethnic groups in sport in the UK at both elite and grassroots level and both men and women’s sports [42].

Although racism is part of the additional stress on migrants, it seems likely that other components (such as social isolation) would be greater in migrants than in other minority ethnic groups. To examine the effect that stress has on the body, some researchers use ‘allostatic load’ which is a physiological measure of the cumulative impact of stress on the body derived through a combination of blood pressure measurements, pulse rate and inflammatory markers in the blood. In a study of allostatic load in US-born and migrant black adults, migrants were found to have reduced allostatic load compared with the US-born [64]. In conclusion, stress as a mediator of unhealthy weight gain in migrant groups is a plausible theory, but without much current evidence.

### Addressing Obesity in Migrant Groups

#### Thresholds for Action

Adult obesity is a risk factor for type 2 diabetes, cardiovascular disease, cancer, osteoarthritis, chronic kidney disease, late-onset Alzheimer’s disease and pregnancy complications [65–67]. It is associated with reduced life expectancy and reduced healthy life expectancy. Child obesity is a risk factor for adult obesity and associated health consequences [68], but is also independently associated with premature mortality, early-onset type 2 diabetes, asthma, musculoskeletal problems, psychological problems and cardiovascular risk factors including hypertension and hypercholesterolaemia [69].

It is highly likely that the same degree of obesity poses a greater health risk for certain ethnic groups; however, determining this empirically is complex. Firstly, common measurements of obesity (such as BMI) may reflect differing accumulations of body fat and in general these may over- or under-predict true obesity [70]. Secondly, even with equal amounts of body fat, where this is distributed and given that abdominal obesity gives rise to greater health risks than subcutaneous obesity, this may result in differing risk. Both these things are likely to be heritable and could vary between ethnic groups [71]. There are even more complications when considering thresholds for childhood obesity, given the influence of different ages of growth and sexual maturation on body composition, which can vary by ethnic group [72–74]. Lastly, even with equal amounts and distribution of body fat, the health consequences of obesity may differ between groups because of underlying differences in physiology (e.g. variations in the blood-circulatory system), culture (e.g. age at which people start a family) and environment (e.g. health care access).

Fifteen years ago, a WHO expert consultation examined whether adult Asian populations have different associations between BMI, percentage body fat and health risks than do European populations [75]. This was done to determine recommended BMI thresholds for overweight and obesity in Asian populations. The WHO consultation concluded that there was wide variation among Asian populations and that the international cutoff points for BMI categories should be retained. However, they also recommended additional, lower trigger points for Public Health action in Asian populations (Table 2). In the UK, in 2013, the National Institute for Health and Care Excellence (NICE) went further by extending the recommended lower thresholds to black African and African-Caribbean populations in the UK [76].

**Table 2:** International guidance on BMI thresholds for adult Asian populations

White European populations	Asian populations	Description
Less than 18.5 kg/m^2^	Less than 18.5 kg/m^2^	Underweight
18.5–24.9 kg/m^2^	18.5–23 kg/m^2^	Increasing but acceptable risk
25–29.9 kg/m^2^	23–27.5 kg/m^2^	Increased risk
30 kg/m^2^ or higher	27.5 kg/m^2^ or higher	High risk

Source: WHO 2004 [69], NICE 2013 [80]

Both sets of recommendations were based on patchy evidence of mixed quality. In particular, the main outcomes considered were type 2 diabetes and cardiovascular disease, and categorisation of ethnic groups was broad. Very little evidence exists on the other health risks associated with obesity, and homogenising ethnic groups may hide important differences between distinct populations. In fact, in a study published since the NICE guidance examining half a million UK Biobank participants, South Asian and Chinese groups were demonstrated to have a similar risk of type 2 diabetes at very different BMI (22 vs 24 kg/m^2^) and waist circumference (79 vs 88 cm), which suggests the amalgamation of these groups in the NICE and WHO guidance as ‘Asian’ may not be appropriate [70]. In addition, the evidence presented at NICE was conflicting on whether lower or higher thresholds were appropriate for black populations, despite the decision to then recommend a lower threshold for individuals within these groups.

Other attempts at quantifying risk based on obesity and ethnicity include a joint scientific statement from a number of US and international bodies, which proposes a definition of metabolic syndrome based on differing thresholds for waist circumference in different populations, some of which correspond to ethnic groups [77]. There have been no attempts to officially recommended thresholds for identifying overweight and obesity in children specific to ethnic background, although unofficial thresholds have been proposed [74].

It is highly likely that different ethnic groups have different levels of health risk, in general, at the same BMI and even at the same percentage body fat, but existing evidence does not adequately quantify this. Best practice is likely to involve using indicators of obesity in the context of other personal information on risk factors when considering clinical action.

#### Specific Interventions

Obesity prevention and treatment interventions are recommended in clinical guidance worldwide e.g. [78, 79]. In terms of migrant populations, a key issue is that there may be a difference in intervention effectiveness in some populations, but there is a lack of representation in studies. For example, a Cochrane review of childhood obesity interventions noted that the majority of research in the field had been conducted with ‘motivated, middle-class, Caucasian populations’ which means it may not be generalizable to other populations groups [80]. Similarly, a systematic review of health promotion interventions for minority ethnic groups found that there is currently a lack of evidence on how best to delivery physical activity or healthy eating interventions in these populations [81]. A recent systematic review of interventions to prevent obesity in US migrant populations identified 20 studies [82•]. Although the majority of the included studies were quasi-experimental and therefore limited in terms of the conclusions that can be drawn, the interventions which showed positive effects on obesity all incorporated some cultural focus [82•].

More recently, in the UK, a culturally appropriate intervention for prevention of childhood obesity in the South Asian population was promising in a feasibility study [83] and is now being examined in a definitive cluster randomised controlled trial [84]. A further obesity treatment intervention for Pakistani and Bangladeshi children is also underway [85].

Taken together, the evidence suggests that culturally adapted obesity interventions can be effective—perhaps through increasing the salience, acceptability and uptake of these interventions by migrant groups. However, the number of obesity prevention and treatment interventions for migrant populations does not reflect the growing and diverse groups of immigrants in HICs, and more evidence-based culturally relevant interventions are needed.

## Methods

We searched MEDLINE and Google scholar in May 2017 to identify relevant literature. This was supplemented with reference searching and the authors’ personal libraries of relevant literature. We aimed to examine relevant systematic reviews from the past 10 years, as well as key studies on predetermined topics from the last 3–5 years in order to complete this narrative review. These topics were based on an agreed framework for the review and included epidemiology of obesity, factors contributing to obesity prevalence and addressing obesity in migrant groups.

### Limitations in the Evidence Base

Being repeated throughout our review, we note a general lack of evidence on migrant or generation status in research on ethnic differences in health and the determinants of health. Where it is considered, amalgamations make it difficult to tease apart effects for distinct migrant groups. Also, this topic is complex and it is difficult to account for the roles of length of time in new country vs cohort effects; specific country of origin; the intersection of religion, ethnicity and migrant status; socio-economic status in country of origin, etc. Patterns of health across migrant groups are likely to be dynamic, not stable, for example depending on factors relating to the social, economic and political environment in home and new country at the time of migration and changes in these environments over time, particularly relating to the nutrition transition. This means that findings from one setting may not be generalizable to others.

## Conclusion

Between 1990 and 2015, the number of international migrants worldwide rose by over 91 million or by 60% [86]. Migrants may arrive in new countries with a health advantage including generally a healthier body weight; however, intrinsic and environmental factors combine to cause unhealthy weight gain, often to beyond the levels seen in native populations. Meeting the challenge of prevention and treatment of obesity in diverse populations will require greater attention to minority groups in research in the future.
